# Pyroptosis-mediator Gasdermin D in serum: a potential biomarker in diabetic kidney disease

**DOI:** 10.3389/fendo.2026.1734036

**Published:** 2026-02-18

**Authors:** Nan Ding, Changmei Wei, Qiong Liu, Xuexin Liu, Lijing Huo, Fang Yu, Chaoju Yang

**Affiliations:** 1 Department of Medical Laboratory, Hebei General Hospital, Shijiazhuang, Hebei, China; 2 Department of Nephrology, Hebei General Hospital, Shijiazhuang, Hebei, China

**Keywords:** biomarker, diabetic kidney disease, Gasdermin D, inflammation, pyroptosis

## Abstract

**Objective:**

To evaluate whether serum Gasdermin D (GSDMD) levels are associated with diabetic kidney disease (DKD) and renal function impairment, and to assess its potential diagnostic value.

**Methods:**

This cross-sectional observational study included 111 patients with DKD, 100 patients with non-diabetic kidney diseases, and 135 healthy controls. Serum GSDMD levels were measured using a chemiluminescence assay. Associations between GSDMD and clinical parameters were analyzed using Spearman correlation and binary logistic regression. Receiver operating characteristic (ROC) curves were constructed to evaluate diagnostic performance.

**Results:**

Serum GSDMD levels were significantly higher in patients with DKD than in healthy controls (P < 0.05). GSDMD levels were positively correlated with glucose (GLU), creatinine (CREA), blood urea nitrogen (BUN), and urinary albumin-to-creatinine ratio (UACR), and negatively correlated with estimated glomerular filtration rate (eGFR) (all P < 0.01). Multivariate logistic regression identified GSDMD as an independent factor associated with DKD. The area under the ROC curve (AUC) for GSDMD in identifying DKD was 0.847 (95% CI: 0.808–0.886), which increased to 0.933 (95% CI: 0.904–0.962) when combined with conventional indicators.

**Conclusion:**

Serum GSDMD levels are significantly associated with diabetic kidney disease and renal dysfunction. These findings suggest that GSDMD may serve as a complementary biomarker for DKD assessment; however, longitudinal and multicenter studies are required to confirm its prognostic value and clinical applicability.

## 1 Introduction

Diabetic kidney disease (DKD) is one of the most debilitating microvascular complications of diabetes mellitus, affecting approximately 20–40% of diabetic patients worldwide. It has emerged as the leading cause of end-stage renal disease (ESRD), imposing a severe socioeconomic burden on global healthcare systems (1, 2). The pathogenesis of DKD is complex, involving a cascade of metabolic dysregulation, oxidative stress, and chronic inflammation, which has been supported by both mechanistic and population-based studies (3–5). While the urinary albumin-to-creatinine ratio (UACR) and estimated glomerular filtration rate (eGFR) are standard diagnostic tools, they exhibit significant limitations in terms of early sensitivity and prognostic specificity (6–8). Thus, there is an urgent need for novel biomarkers that reflect the underlying molecular pathology of DKD.

Recent studies have highlighted the pivotal role of pyroptosis—a pro-inflammatory form of programmed cell death—in the progression of diabetic renal injury. This process is typically initiated via activation of inflammasomes, leading to cleavage of GSDMD by caspases (9). Upon activation by inflammatory caspases (such as caspase-1/4/11), GSDMD is cleaved into an N-terminal fragment that forms large pores in the plasma membrane. This leads to cell lysis and the massive release of pro-inflammatory cytokines, including IL-1β and IL-18 (10, 11). In the diabetic milieu, high glucose levels and advanced glycation end-products (AGEs) trigger GSDMD-mediated pyroptosis in essential renal cells, including podocytes, renal tubular cells, and glomerular endothelial cells, directly contributing to glomerular filtration barrier breakdown and interstitial fibrosis (12). Beyond renal pathology, GSDMD-mediated inflammation is implicated in other diabetic complications such as cardiomyopathy and neuropathic pain (13, 14). In addition, emerging evidence suggests that targeting GSDMD-related pathways may have therapeutic relevance in diabetic kidney disease (15). Despite the robust evidence from experimental models linking GSDMD to renal damage, clinical data evaluating its utility as a circulating biomarker in human DKD remain limited.

In this study, we measured serum GSDMD levels in patients with DKD, other kidney diseases, and healthy individuals. Our primary objective was to evaluate the correlation between serum GSDMD and clinical markers of renal function (e.g., UACR, eGFR) and to assess its potential as a promising biomarker for DKD assessment.

## 2 Materials and methods

### 2.1 Participants

This was a cross-sectional observational study conducted at Hebei General Hospital. The design and reporting of this study adhere to the Strengthening the Reporting of Observational Studies in Epidemiology (STROBE) guidelines. Patient identification through endocrinology and nephrology clinic database screening; conduct purposive sampling based on predetermined criteria; After obtaining informed consent, conduct research on blood samples collected from the included participants during their regular consultations. This represents a convenience sample from a hospital-based patient population. The study protocol was approved by the Ethics Committee of Hebei General Hospital (Approval No. 2024-268), and written informed consent was obtained from all participants.

### 2.2 Inclusion and exclusion criteria

Participants with DKD were eligible if they were aged 18–80 years, had a diagnosis of type 2 diabetes mellitus according to ADA 2020 criteria, and had clinical evidence of DKD based on elevated UACR and/or reduced eGFR. Exclusion criteria included acute or chronic infections, uncontrolled hypertension, use of medications (e.g., RAAS inhibitors, statins), autoimmune diseases, malignancies, recent cardiovascular events, pregnancy, dialysis, or renal transplantation. Healthy controls were matched by age and sex and had no history of hypertension, diabetes, renal disease, or systemic inflammation. Patients with other renal diseases (e.g., lupus nephritis, IgA nephropathy, hypertensive nephropathy) formed a comparator group.

### 2.3 Sample collection and GSDMD detection

All subjects were required to fast for more than 8h. On the next morning, 5 ml of fasting venous blood was withdrawn and coagulated at room temperature for 2h. The supernatant was centrifuged at 1000×g at 4°C for 15 min. Take the appropriate amount of serum into EP tubes and store in a deep freezer at -80°C to avoid repeated freezing and thawing. Glucose (GLU), blood urea nitrogen (BUN), creatinine (CREA), and urinary albumin/creatinine ratio (UACR)were measured using BECKMAN AU5800 automatic biochemical analyzer (Beckman, USA); estimated glomerular filtration rate (eGFR) results were calculated using the Chronic Kidney Disease Epidemiology (CKD-EPI) formula; and GSDMD proteins were determined using a commercially available chemiluminescence assay (Beijing MDTK Biotechnology Co., Ltd.) according to the manufacturer’s instructions. According to the manufacturer’s specifications, the intra-assay coefficient of variation (CV) for the GSDMD kit is <8% and the inter-assay CV is < 15%. The limit of detection (LOD) is 1pg/ml. All serum samples were collected, stored, and analyzed under identical experimental conditions to minimize technical variability.

### 2.4 Statistical analysis

Data distribution was assessed using the Normality Test (Shapiro-Wilk Test). Continuous variables were expressed as mean ± SD or median (IQR) and compared using t-tests or Kruskal–Wallis tests as appropriate. Categorical variables were compared using chi-square tests. Spearman correlation was used to evaluate associations between GSDMD and clinical parameters. Logistic regression models were constructed to identify factors independently associated with DKD. Multicollinearity between independent variables was assessed using the variance inflation factor (VIF), with VIF values < 5 indicating no significant multicollinearity. Diagnostic performance was evaluated using ROC curves, and the area under the curve (AUC) was calculated. The figures were created using GraphPad Prism 8. Statistical analyses were performed with SPSS version 25.0, and significance was set at P < 0.05.

An *a priori* sample size calculation was performed using G*Power software. Based on a one-way ANOVA (fixed effects, omnibus) with three groups, assuming a medium effect size (f = 0.25), an α error probability of 0.05, and a statistical power of 0.80, the minimum required total sample size was estimated to be 159 participants. The final sample size of the present study (n = 346) exceeded this requirement, indicating that the study was adequately powered.

## 3 Results

### 3.1 Comparison of serum GSDMD and other DKD-related indicators in different clinical subgroups

**Figure 1:**
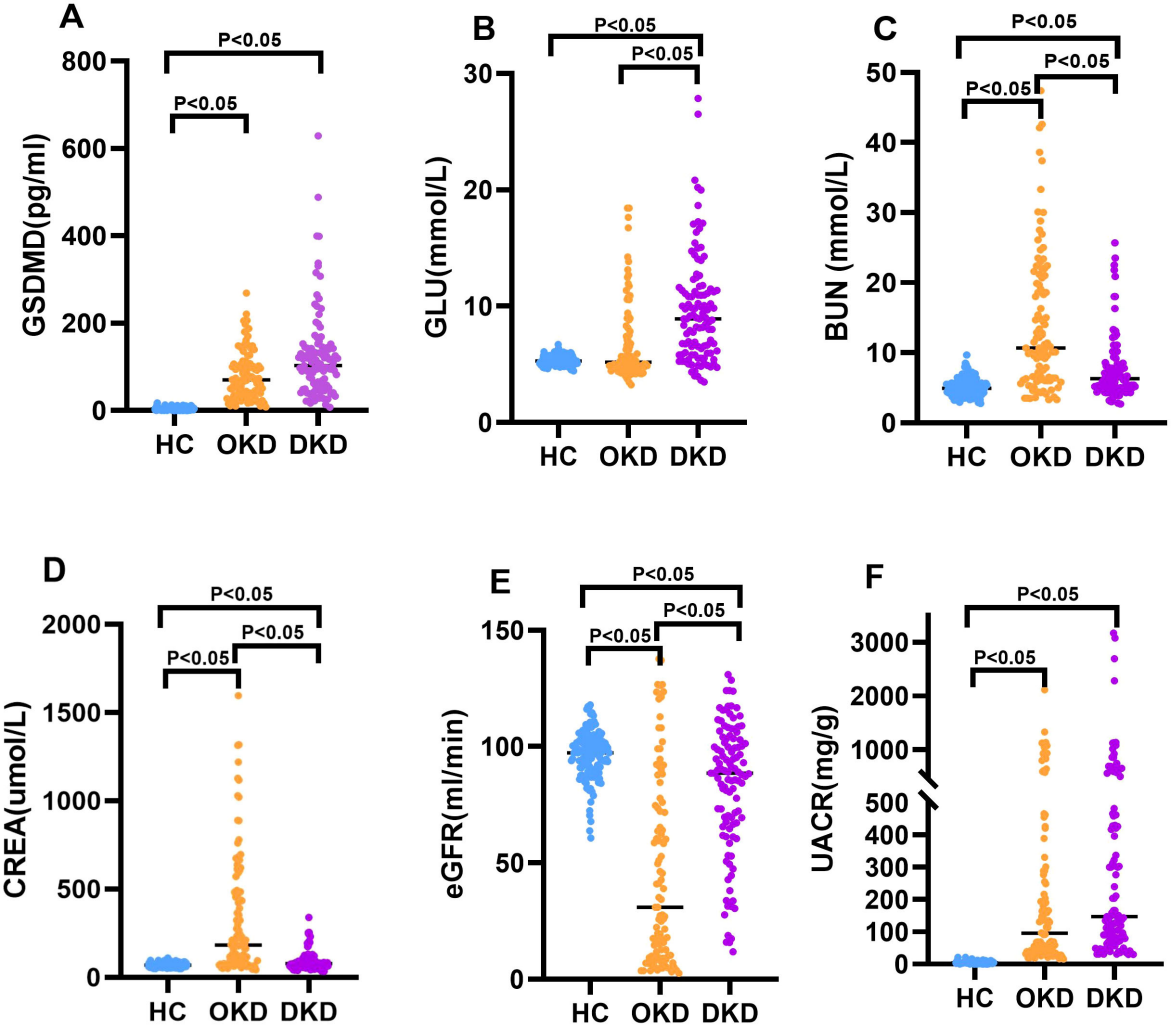
Distribution of serum GSDMD and clinical biomarkers across study groups. Scatterplots showing serum levels of **(A)** Gasdermin D (GSDMD), **(B)** glucose (GLU), **(C)** blood urea nitrogen (BUN), **(D)** creatinine (CREA), **(E)** estimated glomerular filtration rate (eGFR), and **(F)** urinary albumin-to-creatinine ratio (UACR) in healthy controls (HC), patients with other kidney diseases (OKD), and patients with diabetic kidney disease (DKD). Each dot represents an individual participant, and horizontal lines indicate median values. Data were analyzed using the Kruskal–Wallis test followed by pairwise comparisons. P < 0.05 was considered statistically significant. For UACR, a broken y-axis was applied to improve visualization of data distribution.

This study included 111 patients with diabetic kidney disease, 100 patients with other nephropathies and 135 healthy controls. There was no statistically significant difference in age, male/female ratio and Duration of disease (years) among the three groups (P>0.05); The differences in GLU, BUN, CREA, eGFR and UACR among the three groups were statistically significant (P<0.05). The serum levels of GSDMD in each group were 103.10 (51.04, 142.93) pg/ml in the diabetic kidney disease group; 70.20 (34.88,107.47) pg/ml in the other kidney disease group; and 3.38 (1.42,5.67) pg/ml in the healthy control group. The difference between the diabetic kidney disease group and the healthy control group was statistically significant (P< 0.05), and diabetic kidney disease group was higher than other kidney disease groups, but the difference was not statistically significant (P>0.05) (**Table 1**; **Figure 1**).

**Table 1:** Comparison of each clinical data and serum GSDMD between the three groups.

Project	Healthy control group (N=135)	Other kidney disease group (N=100)	Diabetic kidney disease group (N=111)	X2/F/H/U	P value
Age (year)	57.84 ± 8.90	57.44 ± 12.79	60.15 ± 14.20	1.46	P=0.18
Gender(female/male)	83/52	55/45	74/37	4.06	P=0.13
Duration of disease (years)	0	11.35(5.63,15.20)	10.00(4.90,13.90)	6175.5	P=0.16
GSDMD (pg/ml)	3.38(1.42,5.67)	70.20(34.88,107.47) ♦	103.11(51.04, 142.93)♦	249.24	P<0.01
GLU(mmol/L)	5.29(5.07,5.61)	5.18(4.64,7.49)*	8.90(5.96,11.37)♦	79.51	P<0.01
BUN (mmol/L)	4.93(4.07,5.89)	10.70(6.13,20.50) ♦*	6.30(4.90,8.00)♦	96.03	P<0.01
CREA(umol/L)	68.90(59.10,77.50)	181.90(73.83,483.60) ♦*	76.80(59.90,100.50)♦	83.48	P<0.01
eGFR(ml/min)	97.34(89.60,102.67)	30.93(9.30,75.69) ♦*	88.59(66.50,103.35)♦	89.54	P<0.01
UACR(mg/g)	14.34(7.65,25.54)	96.09(35.96,289.07) ♦	146.92(69.57,459.33)♦	203.67	P<0.01

GSDMD, gasdermin D; GLU, glucose; BUN, blood urea nitrogen; CREA, creatinine; eGFR, estimated glomerular filtration rate; UACR, urinary albumin/creatinine ratio; ♦Comparison with healthy control group P<0.05; *Comparison with DKD group P<0.05; GSDMD, GLU, BUN, CREA, eGFR, and UACR are expressed as median (interquartile range), comparisons between groups were performed using Kruskal-Wallis test; Age is expressed as mean ± standard deviation, comparisons between groups were performed using t-tests; gender comparisons between groups were performed using chi-square tests; Duration of disease (years) are expressed as median (interquartile range), comparisons between two disease groups were performed using Mann-Whitney U test.

### 3.2 Correlation between serum GSDMD and clinical parameters of DKD

**Figure 2:**
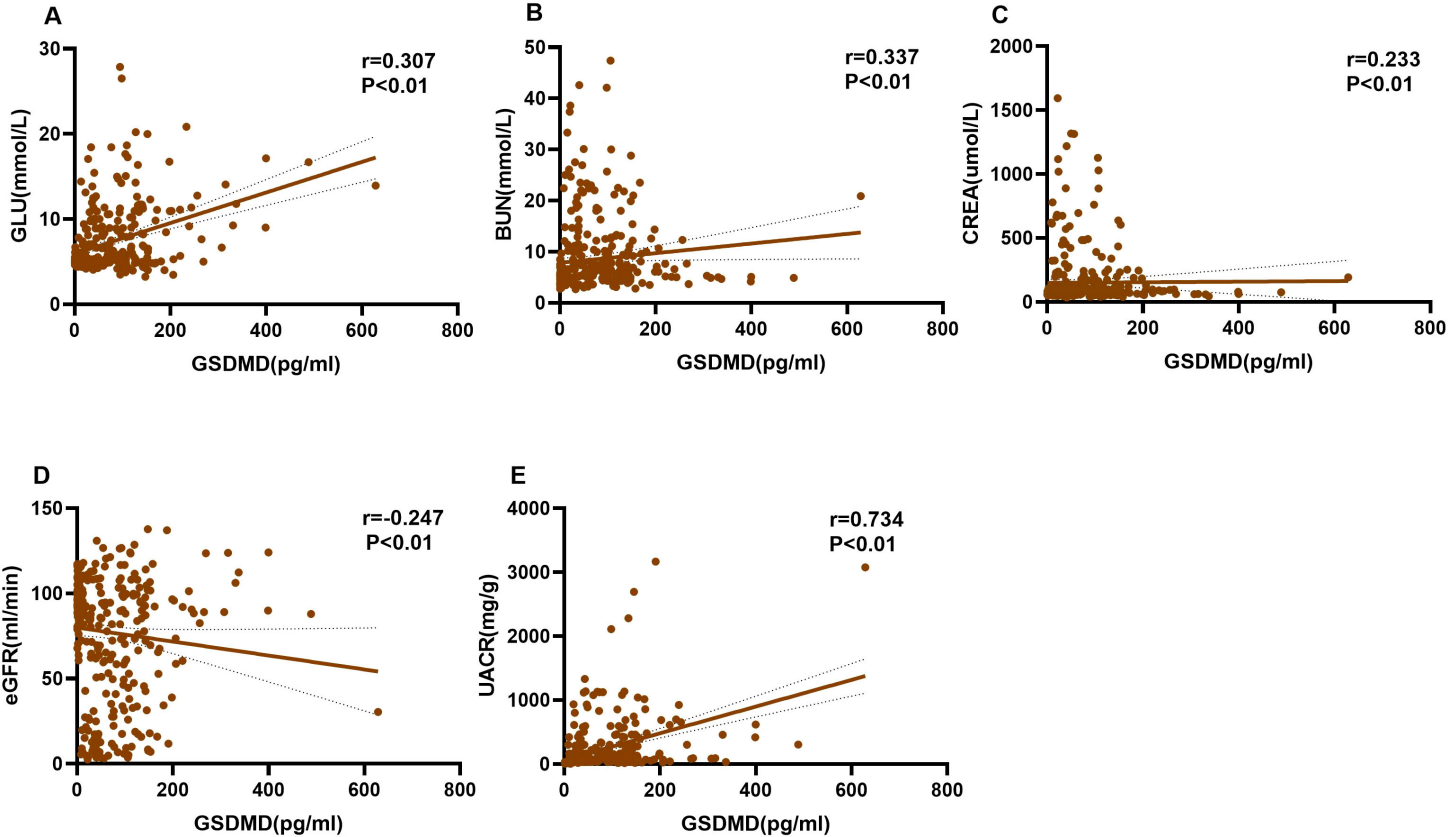
Correlations between serum GSDMD levels and clinical parameters. Scatterplots showing the correlations between serum GSDMD levels and **(A)** glucose (GLU), **(B)** blood urea nitrogen (BUN), **(C)** creatinine (CREA), **(D)** estimated glomerular filtration rate (eGFR), and **(E)** urinary albumin-to-creatinine ratio (UACR). Spearman’s rank correlation analysis was performed among all study participants. Solid lines represent fitted regression trends, and dotted lines indicate 95% confidence intervals. The r represents the Spearman correlation coefficient. P < 0.01 indicates high statistical significance.

The serum GSDMD level was positively correlated with GLU (r = 0.307, P < 0.01), CREA (r = 0.233, P < 0.01), BUN (r = 0.337, P < 0.01), and UACR (r = 0.734, P < 0.01), and negatively correlated with eGFR (r = -0.247, P < 0.01) (**Table 2**; **Figure 2**).

**Table 2:** Spearman correlation analysis between serum GSDMD and clinical parameters.

Project	Serum GSDMD	
	r	P value
GLU(mmol/L)	0.307	<0.01
BUN(mmol/L)	0.337	<0.01
CREA(umol/L)	0.233	<0.01
eGFR(ml/min)	-0.247	<0.01
UACR(mg/g)	0.734	<0.01

GSDMD, gasdermin D; GLU, glucose; BUN, blood urea nitrogen; CREA, creatinine; eGFR, estimated glomerular filtration rate; UACR, urinary albumin/creatinine ratio, r values represent Spearman correlation coefficients.

### 3.3 Univariate binary logistic regression analysis of all parameters

A univariate binary logistic regression analysis was performed with the occurrence of DKD as the dependent variable (assignment: DKD = 1, non-DKD=0, N = 346) and GSDMD, GLU, BUN, CREA, eGFR, UACR as the independent variables. The results showed that GSDMD, GLU, CREA, and UACR may be potential influencing factors for the occurrence of DKD (P < 0.05) (**Table 3**).

**Table 3:** Univariate binary logistic regression analysis of all parameters.

Project	P value	OR		95% CI
GSDMD (pg/ml)	P<0.01		1.019	1.014~1.024
GLU (mmol/L)	P<0.01		1.411	1.285~1.550
CREA (umol/L)	P<0.01		0.996	0.993~0.999
UACR (mg/g)	P<0.01		1.002	1.001~1.003

GSDMD, gasdermin D; GLU, glucose; BUN, blood urea nitrogen; CREA, creatinine; eGFR, estimated glomerular filtration rate; UACR, urinary albumin/creatinine ratio, The reference group for the dependent variable was “Non-DKD group” (N=346).

### 3.4 Multivariate binary logistic regression analysis of serum GSDMD

A multivariate binary logistic regression analysis was performed with the occurrence of DKD as the dependent variable (assignment: DKD = 1, non-DKD=0) and GSDMD, GLU, CREA, UACR as the independent variables. The results showed that serum GSDMD may be a potential influencing factor for the clinical occurrence of DKD (P < 0.05). After adjusting for the three factors of GLU, CREA, and UACR, it was found that GSDMD is an independent risk factor for the occurrence of DKD (**Table 4**).

**Table 4:** Multivariate binary logistic regression analysis of all parameters.

Project	P value	OR		95% CI
GSDMD (pg/ml)	P<0.01		1.016	1.010~1.022
GLU (mmol/L)	P<0.01		1.374	1.215~1.553
CREA (umol/L)	P<0.01		0.979	0.970~0.988
UACR (mg/g)	P<0.01		1.005	1.003~1.006
constant	–		0.065	–

GSDMD, gasdermin D; GLU, glucose; BUN, blood urea nitrogen; CREA, creatinine; eGFR, estimated glomerular filtration rate; UACR, urinary albumin/creatinine ratio, The reference group for the dependent variable was “Non-DKD group” (N=346). Collinearity diagnostics were performed, and all Variance Inflation Factor (VIF) values were less than 5, indicating no significant collinearity.

### 3.5 Diagnostic performance of GSDMD

**Figure 3:**
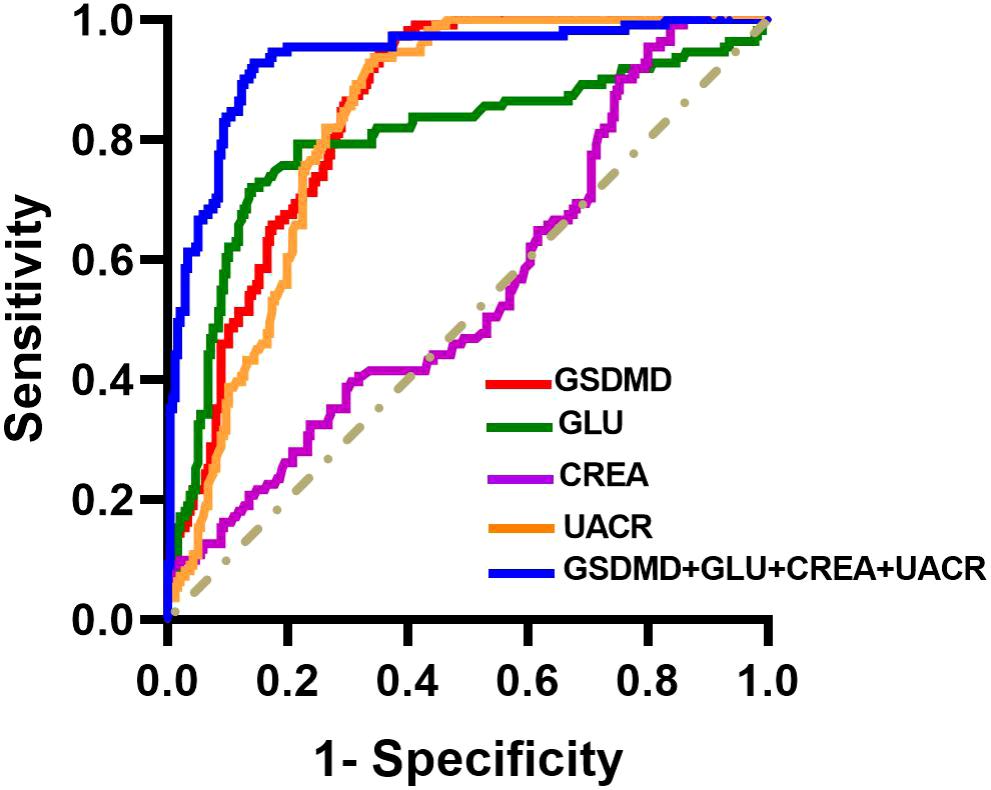
Receiver operating characteristic (ROC) curves for the diagnostic performance of serum GSDMD in diabetic kidney disease. ROC curves illustrating the diagnostic performance of serum GSDMD, glucose (GLU), creatinine (CREA), urinary albumin-to-creatinine ratio (UACR), and the combined model in distinguishing patients with diabetic kidney disease from non-DKD controls. The area under the curve (AUC) and corresponding 95% confidence intervals are provided in **Table 5**.

We defined the occurrence of DKD as the case group and assessed the diagnostic performance of serum GSDMD, GLU, CREA, and UACR based on the area under the receiver operating characteristic curve (AUC). The AUC for GSDMD in identifying DKD was 0.847 (95% CI: 0.808, 0.886), with an optimal cutoff value of 21.32 pg/mL within this study cohort, yielding a sensitivity of 97.30% and a specificity of 62.13%. The combined use of GSDMD with GLU, CREA, and UACR improved the diagnostic performance, yielding an AUC of 0.933 (95% CI: 0.904, 0.962) (**Table 5**; **Figure 3**).

**Table 5:** Diagnostic performance of GSDMD.

Subjects	AUC	P value	95%CI	Optimal cut-off value	Sensitivity%	Specificity%	Youden index
GSDMD(pg/ml)	0.847	<0.01	0.808~0.886	21.32	97.30	62.13	0.59
GLU (mmol/L)	0.797	<0.01	0.740~0.853	6.33	72.10	86.00	0.58
CREA (umol/L)	0.450	0.136	0.386~0.515	75.85	52.30	53.62	0.05
UACR (mg/g)	0.827	<0.01	0.778~0.863	34.30	93.69	65.53	0.57
GSDMD +GLU +CREA+UACR	0.933	<0.01	0.904~0.962	–	92.80	85.53	0.78

GSDMD, gasdermin D; GLU, glucose; BUN, blood urea nitrogen; CREA, creatinine; eGFR, estimated glomerular filtration rate; UACR, urinary albumin/creatinine ratio, The optimal cut-off value was determined by maximizing the Youden’s index.

### 3.6 Relationship between GSDMD and DKD progression

**Figure 4:**
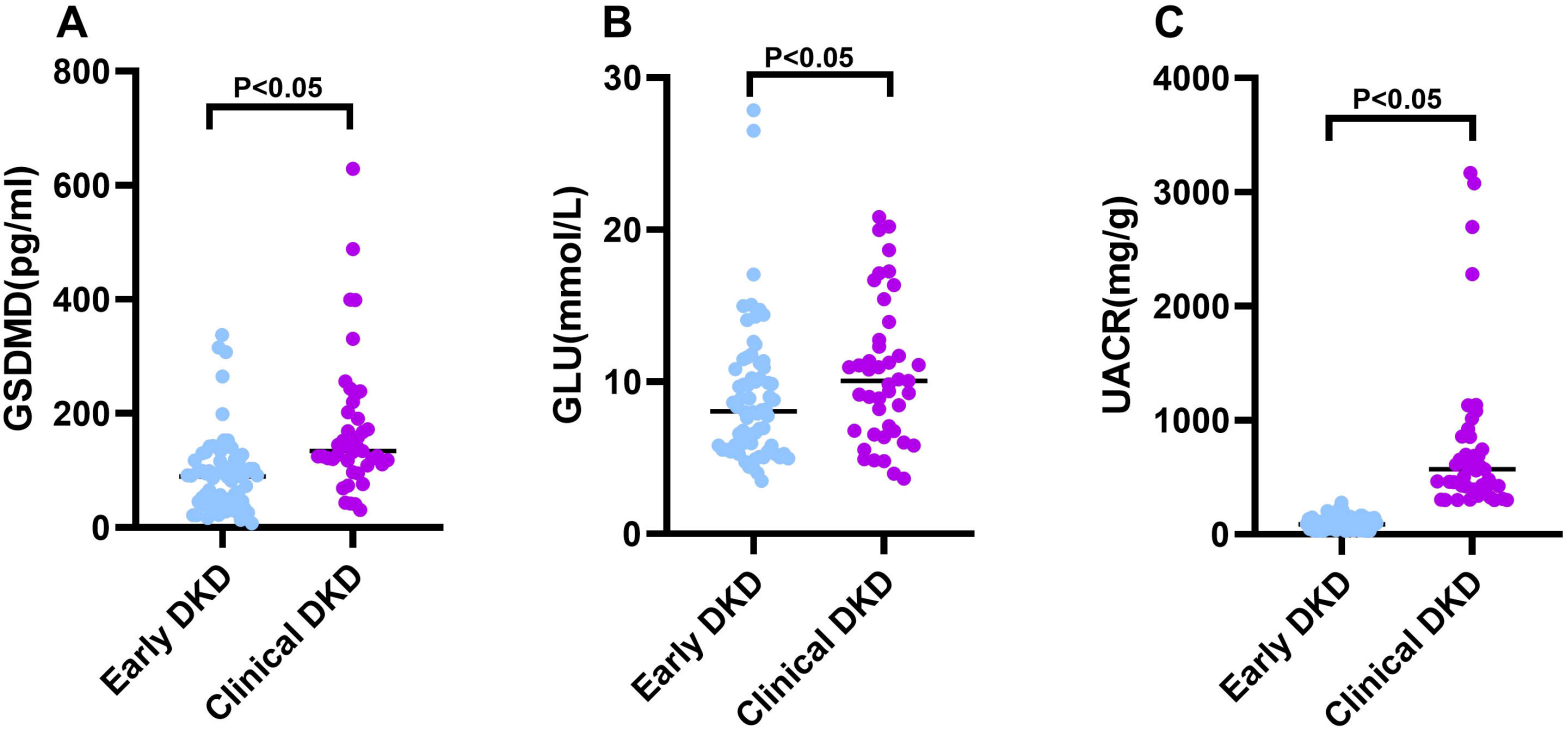
Comparison of serum GSDMD and clinical parameters according to DKD severity. Scatterplots showing the distribution of **(A)** Gasdermin D (GSDMD), **(B)** glucose (GLU), and **(C)** urinary albumin-to-creatinine ratio (UACR) in patients with early diabetic kidney disease (UACR 30–300 mg/g) and clinical diabetic kidney disease (UACR ≥ 300 mg/g). Each dot represents an individual participant, and horizontal lines indicate median values. Statistical comparisons were performed using the Mann–Whitney U test. P < 0.05 was considered statistically significant.

Using UACR as a criterion, 30 mg/g ≤ UACR ≤ 300 mg/g was defined as early DKD group, and UACR ≥ 300 mg/g was defined as clinical DKD group. The statistical results showed that the GSDMD in the clinical DKD group was significantly higher than that in the early DKD group (P<0.01), and the results indicated that the GSDMD increased gradually with disease progression and was associated with disease progression (**Table 6**; **Figure 4**).

**Table 6:** Comparison of serum GSDMD and clinical indicators under different disease progression subgroups in DKD.

Project	Early DKD group	Clinical DKD group	P value
Age (year)	59.90 ± 15.36	60.56 ± 12.31	P=0.812
Gender(female/male)	47/21	27/16	P=0.306
GSDMD (pg/ml)	89.40(53.63,143.28)	134.10(110.83,202.29) ▲	P<0.01
GLU (mmol/L)	8.08(5.62, 10.91)	10.05(6.75,12.76) ▲	P<0.05
BUN (mmol/L)	5.65(4.80, 7.65)	6.60(5.10,11.10)	P=0.065
CREA (umol/L)	73.40(59.93,97.70)	78.40(59.90, 108.70)	P=0.263
eGFR (ml/min)	89.10(70.62,103.87)	87.91(60.39, 101.30)	P=0.191
UACR (mg/g)	86.94(47.89,136.67)	571.15(417.87,859.34) ▲	P<0.01

GSDMD, gasdermin D;GLU, glucose; BUN, blood urea nitrogen; CREA, creatinine; eGFR, estimated glomerular filtration rate; UACR, urinary albumin/creatinine ratio; ▲Comparison with early DKD group P<0.05. GSDMD, GLU, BUN, CREA, eGFR, and UACR are expressed as median (interquartile range), comparisons between groups were performed using Mann-Whitney test; Age is expressed as mean ± standard deviation, comparisons between groups were performed using t-tests; gender comparisons between groups were performed using chi-square tests.

## 4 Discussion

In this cross-sectional observational study, we identified significantly elevated serum GSDMD levels in patients with DKD. Notably, circulating GSDMD concentrations correlated closely with well-established renal injury biomarkers, including UACR, CREA, BUN, and eGFR, and increased progressively with DKD severity. A key finding of this study is the strong association between serum GSDMD and UACR, which underscores a direct link between GSDMD and albuminuria—a hallmark of glomerular barrier dysfunction and a pivotal predictor of DKD progression. The observed relationship implies that GSDMD may serve as a surrogate indicator of ongoing inflammatory cell injury and structural renal impairment in DKD. Moreover, the inverse correlation between GSDMD levels and eGFR corroborates its association with renal function deterioration, further reinforcing the utility of serum GSDMD in stratifying DKD severity and evaluating disease status. Importantly, our data suggest that the association between GSDMD and renal impairment is particularly pronounced within the inflammatory milieu characteristic of diabetes. Although the non-DKD group exhibited more severe reductions in eGFR and higher CREA levels, serum GSDMD concentrations remained lower than those observed in DKD patients. This observation implies that GSDMD elevation is not merely a consequence of reduced filtration capacity but is closely linked to diabetes-associated inflammation.

Multivariate logistic regression analysis further validated serum GSDMD as an independent factor associated with DKD, supporting its potential as a clinically relevant biomarker for renal injury in diabetic patients. Our study provides novel insights by demonstrating the independent association of serum GSDMD with DKD. While GSDMD exhibited moderate specificity (62.13%), its high sensitivity (97.30%) underscores its value as a potent ‘rule-out’ screening tool to prevent missed diagnoses. The diagnostic performance of GSDMD (AUC: 0.847) was notable, and its combination with traditional indicators yielded a significantly higher AUC of 0.933. This demonstrates that GSDMD provides incremental diagnostic value as a vital complement to existing markers, reflecting the early inflammatory and pyroptotic damage central to DKD progression.

Our findings are consistent with recent experimental studies linking pyroptosis to diabetic kidney injury. GSDMD is a well-established executor of pyroptosis, activated via cleavage by inflammatory caspases such as caspase-1 and caspase-11 (16, 17). The cleaved GSDMD-N fragment forms membrane pores, leading to cell lysis and release of proinflammatory mediators like IL-1β and IL-18 (10, 11). In the context of DKD, high glucose levels, advanced glycation end-products (AGEs), and oxidative stress can activate inflammatory signaling pathways, including the NLRP3 inflammasome, resulting in caspase-1 activation and subsequent GSDMD-mediated pyroptosis in renal cells such as podocytes and renal tubular epithelial cells (12, 18).This GSDMD-mediated pyroptosis contributes to the structural and functional deterioration of glomeruli and renal tubules, promoting the initiation and progression of DKD (11). Our study shows that serum GSDMD concentrations are significantly elevated in DKD patients and correlate with key renal function parameters. Consistently, a recent prospective cohort study reported that circulating GSDMD levels were increased in uremic patients and were associated with disease prognosis, supporting the clinical relevance of serum GSDMD as a marker of renal injury severity (19).

Moreover, the potential of GSDMD as a therapeutic target in DKD is increasingly recognized. For example, dapagliflozin has been shown to attenuate skeletal muscle atrophy in diabetic nephropathy mice by suppressing GSDMD-mediated canonical pyroptosis, highlighting its role beyond that of a biomarker (15). In parallel, upstream metabolic regulators such as AMPK have been proposed as therapeutic targets in diabetic nephropathy due to their capacity to modulate inflammatory and cell death pathways, including pyroptosis, further supporting the translational relevance of targeting GSDMD-associated signaling networks in DKD (20).

Several limitations of this study should be acknowledged. First, due to the cross-sectional design, a definitive causal relationship between elevated serum GSDMD levels and the development or progression of diabetic kidney disease cannot be established. Consequently, these findings demonstrate a clinical association rather than causality; longitudinal studies are required to determine whether GSDMD elevation precedes the decline in renal function. Second, this was a single-center, hospital-based study employing a convenience sampling strategy, which may limit the generalizability of the results to broader populations, including different ethnic groups and clinical settings. Potential selection bias inherent to this study design cannot be fully excluded. Third, the diagnosis of DKD was based on established clinical criteria rather than renal biopsy confirmation, which might allow for overlap with other subclinical or mixed renal pathologies. Furthermore, upstream inflammasome components and downstream cytokines were not measured, limiting detailed mechanistic insight into the molecular pathways involved. Therefore, prospective studies on pyroptosis-related signaling pathways are warranted to further elucidate these mechanisms. Although an *a priori* sample size calculation indicated adequate statistical power, larger multicenter prospective studies are still warranted to validate these findings and to assess the prognostic value of serum GSDMD. Finally, serum GSDMD was measured using a chemiluminescence-based assay without direct methodological comparison to ELISA, meaning absolute concentration values should be interpreted with caution.

Despite these limitations, our study provides preliminary clinical evidence supporting the association between serum GSDMD and diabetic kidney disease, offering a foundation for future multicenter validation.

## 5 Conclusion

In summary, this study demonstrates that serum Gasdermin D (GSDMD) levels are markedly elevated in patients with diabetic kidney disease and independently correlate with established renal injury markers, including UACR and eGFR. These findings suggests that GSDMD may serve as a sensitive complementary biomarker for assessing inflammatory kidney injury in diabetes, with potential utility for early detection and risk stratification. Although this study is cross-sectional and single-center, and causality cannot be established, it provides a strong foundation for prospective, multicenter investigations to validate the prognostic and mechanistic relevance of GSDMD in DKD.

## Data Availability

The original contributions presented in the study are included in the article/supplementary material. Further inquiries can be directed to the corresponding author.
